# The prognosis of infective endocarditis treated with biological valves versus mechanical valves: A meta-analysis

**DOI:** 10.1371/journal.pone.0174519

**Published:** 2017-04-13

**Authors:** Ende Tao, Li Wan, WenJun Wang, YunLong Luo, JinFu Zeng, Xia Wu

**Affiliations:** 1 Department of Cardiovascular Surgery of the First Affiliated Hospital of Nanchang University, Nanchang, Jiangxi, China; 2 Department of Neurosurgery of the First Affiliated Hospital of Nanchang University, Nanchang, Jiangxi, China; Universita degli Studi di Bologna, ITALY

## Abstract

**Objective:**

Surgery remains the primary form of treatment for infective endocarditis (IE). However, it is not clear what type of prosthetic valve provides a better prognosis. We conducted a meta-analysis to compare the prognosis of infective endocarditis treated with biological valves to cases treated with mechanical valves.

**Methods:**

Pubmed, Embase and Cochrane databases were searched from January 1960 to November 2016.Randomized controlled trials, retrospective cohorts and prospective studies comparing outcomes between biological valve and mechanical valve management for infective endocarditis were analyzed. The Newcastle-Ottawa Scale(NOS) was used to evaluate the quality of the literature and extracted data, and Stata 12.0 software was used for the meta-analysis.

**Results:**

A total of 11 publications were included; 10,754 cases were selected, involving 6776 cases of biological valves and 3,978 cases of mechanical valves. The all-cause mortality risk of the biological valve group was higher than that of the mechanical valve group (HR = 1.22, 95% CI 1.03 to 1.44, *P* = 0.023), as was early mortality (RR = 1.21, 95% CI 1.02 to 1.43, *P* = 0.033). The recurrence of endocarditis (HR = 1.75, 95% CI 1.26 to 2.42, *P* = 0.001), as well as the risk of reoperation (HR = 1.79, 95% CI 1.15 to 2.80, *P* = 0.010) were more likely to occur in the biological valve group. The incidence of postoperative embolism was less in the biological valve group than in the mechanical valve group, but this difference was not statistically significant (RR = 0.90, 95% CI 0.76 to 1.07, *P* = 0.245). For patients with prosthetic valve endocarditis (PVE), there was no significant difference in survival rates between the biological valve group and the mechanical valve group (HR = 0.91, 95% CI 0.68 to 1.21, *P* = 0.520).

**Conclusion:**

The results of our meta-analysis suggest that mechanical valves can provide a significantly better prognosis in patients with infective endocarditis. There were significant differences in the clinical features of patients receiving a biological valve compared to patients receiving a mechanical valve. A large, multicenter retrospective study included in our meta-analysis suggested that any mortality risk of the biological valve group was significant higher than that of the mechanical valve group. However, the risk was no different after risk was adjusted. So, we thought the reason for this result may be related to the characteristics of the patient rather than valve dysfunction. It is still necessary to future randomized studies to verify this conclusion.

## Introduction

Despite improvements in managements, IE remains a deadly disease associated with an in-hospital mortality of 10–30%, and 50% of patients required cardiac surgery during the acute phase[1,2]. The two primary objectives of surgery are the complete removal of the infected tissue and reconstruction of cardiac morphology, including repair or replacement of the affected valves[3]. During the operation, the type of valve including biological valve, mechanical valve, autograft and homograft is selected[4].The 2014 American College of Cardiology/American Heart Association(ACC/AHA) guidelines recommended a biological valve in patients 65 years of age or older, while a mechanical valve is suitable for patients under 65 years of age, but the guidelines do not provide specific recommendations for surgery for IE[5]. The Task Force for the Management of Infective Endocarditis of the 2015 European Society of Cardiology (ESC) does not support any specific valve substitute but recommends a tailored approach for each individual patient and clinical situation[3]. Guidelines from the STS[6] recommend the following: (1)When surgery is indicated for native aortic valve endocarditis, a mechanical or stented tissue valve is acceptable, if the infection is limited to the native aortic valve or to the aortic annulus. Valve choice should be based upon age, life expectancy, comorbidities, and compliance with anticoagulation therapy (Class IIa, Level of evidence B); (2) When surgery is indicated for prosthetic valve aortic endocarditis, it is reasonable to implant a mechanical or stented tissue valve (Class IIa, Level of evidence B). A homograft may be beneficial in aortic valve prosthetic endocarditis when a periannular abscess or extensive destruction of anatomic structures has occurred (Class IIa, Level of evidence B). The choice of valve remains controversial. Some studies[7,8,9,10,11] have found no significant differences in survival between biological valves and mechanical valves, but there are also some studies[4,12] reporting that the survival rate with biological valves is inferior to mechanical valves. Presently, there are no randomized controlled trials or meta-analysis studies compare the prognosis of biological valves with that of mechanical valves. We performed a meta-analysis with available evidence to analyze the prognosis of IE patients treated with biological and mechanical valves to assist clinicians in the selection of valve type.

## Materials and methods

### Literature search

We performed an electronic search with the terms“endocarditis”and“prosthesis”or“mechanical valve”or“biological valve”or “bioprosthetic valve”or“tissue valve”, using Pubmed, Embase and Cochrane databases for studies published between January 1960 and November 2016, comparing biological valves with mechanical valves for IE. We also reviewed the references of listed in original literature to identify possible additional studies. To evaluate the quality of studies, we used the NOS. We assigned the studies of superior quality a score of 9 stars, and high quality studies were assigned a score≥6 stars. The results are shown in Table 1.

**Table 1:** Characteristics of studies included in the meta-analysis.

Study	Country	Study Design	n	B	M	Age(yr)		B survival rate(%)			M survival rate(%)			Study quality
						B	M	1year 3year 5year			1year 3year 5year			
Kim et al.(2016)[26]	America	Prospective cohort	218	139	79	59.8±14.6	47.2±14.5	-	-	-	-	-	-	*******
Delahaye et al.(2015)[4]	America	Prospective cohort	1467	550	917	62	44.8	74.7	-	-	83.4	-	-	********
Savage et al.(2014)[11]	America	Retrospective cohort	7540	5396	2144	57	44.8	-	-	-	-	-	-	*******
Greason et al.(2014)[7]	America	Retrospective cohort	39	16	23	-	-	68.1	55.78	37.80	73.36	64.64	55.54	*******
Nguyen et al.(2010)[12]	France	Prospective cohort	140	31	109	63.2±13.6	57.3±11.6	65.83	51.85	46.31	85.38	81.43	78.21	********
Musci et al.(2010)[8]	Germany	Retrospective cohort	122	93	29	-	-	55.9	-	34.2	44.8	-	42.2	*******
Fedoruk et al.(2009)[9]	England	Retrospective cohort	357	189	169	51.6±17.3	45.6±13.5	-	-	-	-	-	-	*******
Moon et al.(2001)[10]	America	Retrospective cohort	286	221	65	-	-	80.35	71.24	68.33	87.62	80.50	70.83	********
Edwards et al.(1998)[29]	England	Retrospective cohort	322	53	269	-	-	71.3	-	56.3	66.2	-	54.9	*******
Wos et al.(1996)[28]	Poland	Retrospective cohort	71	17	54	-	-	-	-	-	-	-	-	*******
Reul et al.(1989)[30]	America	Retrospective cohort	185	88	97	49	46.6	94.9	83.77	72.75	93.3	88.2	88.2	*******

B: biological valve group; M: mechanical valve group.

### Eligibility criteria

Studies with the following criteria were eligible for inclusion: (1) a randomized controlled trial, retrospective or prospective observational study; (2) patients with definitive diagnosis of infective endocarditis and surgical implantation of biological or mechanical valves; (3) publication dated between January 1960 and November 2016, including HR and 95% CI or Kaplan-Meier curves or other related data.

### Definitions

All-cause mortality was defined as death due to any cause at follow-up. Early mortality was defined as any death that occurred within 30 days after operation or in-hospital. The definition of infective endocarditis according to the modified Duke criteria was used[13].

### Data collection

All the data were extracted independently from two reviewers (EDT and WJW). Any initial disagreement was reviewed and resolved by consensus. The background and characteristics of research (author, publication time, age of patients, study design, number of cases, mortality, survival rate, incidence of recurrence, and incidence of postoperative embolic events) were extracted from each study. HR and 95% CI were obtained directly from the literature or through the effective data calculation and Kaplan-Meier curve extraction. The method of data calculation and Kaplan-Meier curve extraction was similar to the study by Tierney in 2007 [14].

### Outcome

The primary end point was mortality. The secondary end point included in-hospital death or 30-day mortality, embolic events, reoperation and recurrence of infective endocarditis.

### Statistical analysis

The risk ratio (HR) or relative risk (RR) was used as the combined effect, and the effects were expressed as 95% confidence interval (CI). Stata 12.0 software was used in the data analysis and synthesis. Heterogeneity analysis was performed by Cochrane’s Q statistic test for all the clinical trials. I^2^ values of 25%, 50% and 75% were considered as low, moderate and high heterogeneity, respectively. If the heterogeneity of each study was low or *P* > 0.1, the fixed effect model was used; if the heterogeneity of each study was high or *P* < 0.1, the random effects model was used. Meta sensitivity analysis and subgroup analysis were used to explore the sources of heterogeneity between studies. The funnel plot and Egger regression test were used to evaluate publication bias. A *P* value of <0.05 was considered statistically significant.

## Results

### Study characteristics

**Fig 1:**
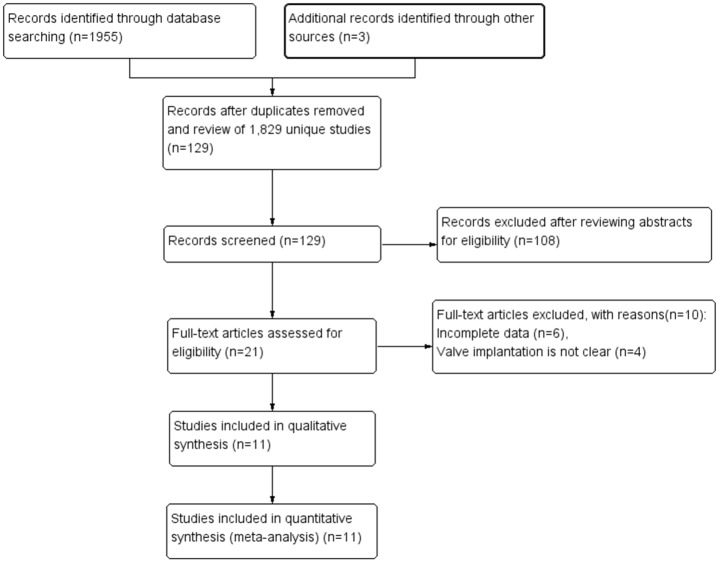
PRISMA flowchart.

As shown in the flow chart (Fig 1), the search strategy initially included 1958 publications. After the title and abstracts were retrieved, 1829 publications were excluded. 129 articles were reviewed in detail. Finally, 11 publications that met the eligibility criteria, which was determined after reading the article. A total of 10,754 patients with IE, including 6,776 cases in the biological valve group and 3,978 cases in the mechanical valve group. Basic information is shown in Table 1 and S2 Table.

### Meta-analysis results

#### All-cause mortality

**Fig 2:**
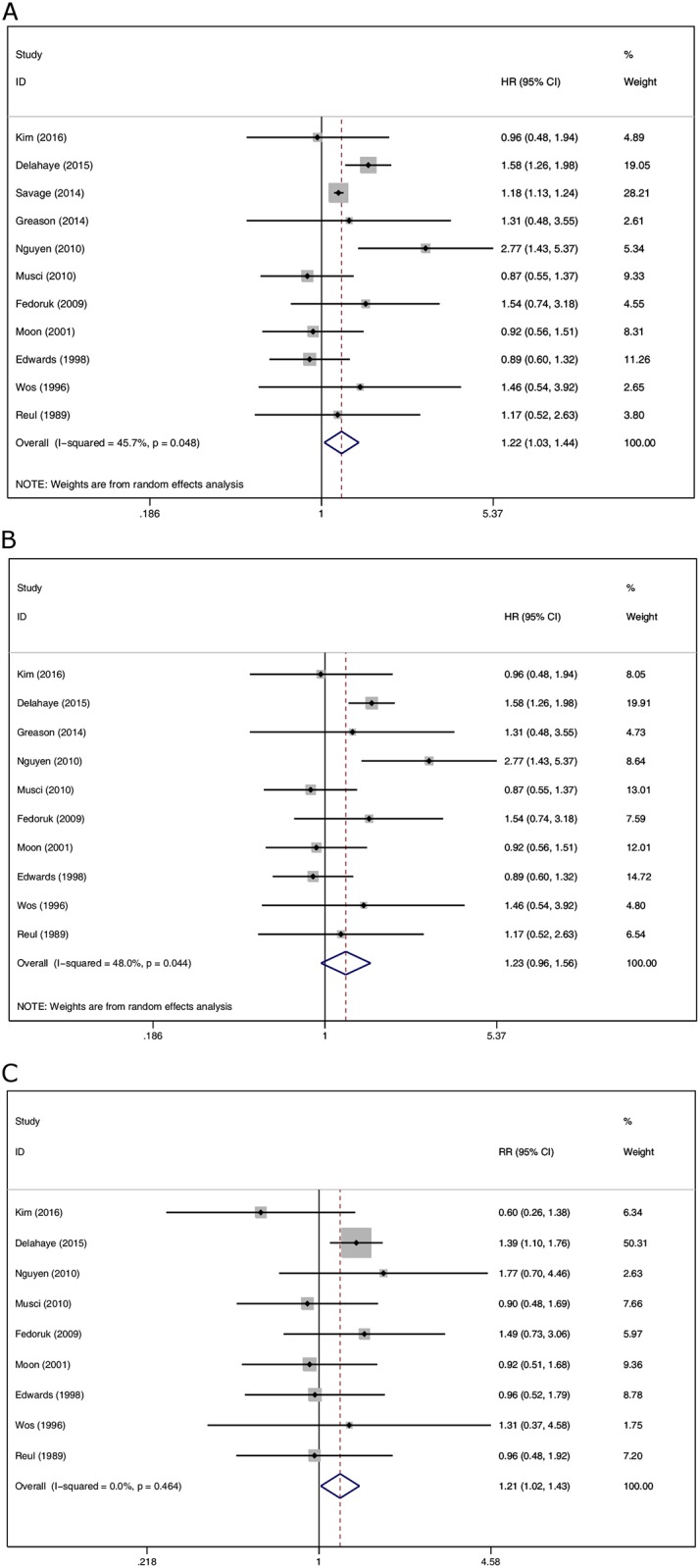
(A) Comparison of all-cause mortality between biological valves and mechanical valves. (B) Exclusion of the study with maximum weight altered the results.(C) Comparison of early mortality between biological valves and mechanical valves.

All-cause mortality was mentioned in 11 studies. In the biological valve group, the risk of death was significantly higher than that in the mechanical valve group (HR 1.22, 95% CI 1.03 to 1.44, *P* = 0.023) (Fig 2A). Heterogeneity was moderate among the included studies (I^2^ = 45.7%), and therefore the random effects model was used. A sensitivity analysis excluding the study[11] with maximum weight altered the results (HR 1.23, 95% CI 0.96 to 1.56, P = 0.099) (Fig 2B).

#### Early mortality

An analysis of the nine studies, including the date of early mortality, showed significantly higher RR of early mortality in the biological valve group (RR 1.21, 95% CI 1.02 to 1.43, *P* = 0.033, I^2^ = 0.0%) (Fig 2C).

#### Subgroup analysis

**Fig 3:**
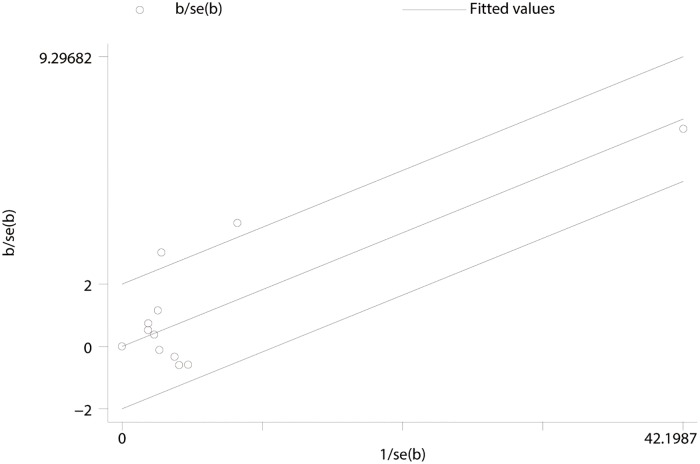
Galbraith plot results indicated studies of Delahaye et al. and Nguyen et al. were located outside the interval.

**Fig 4:**
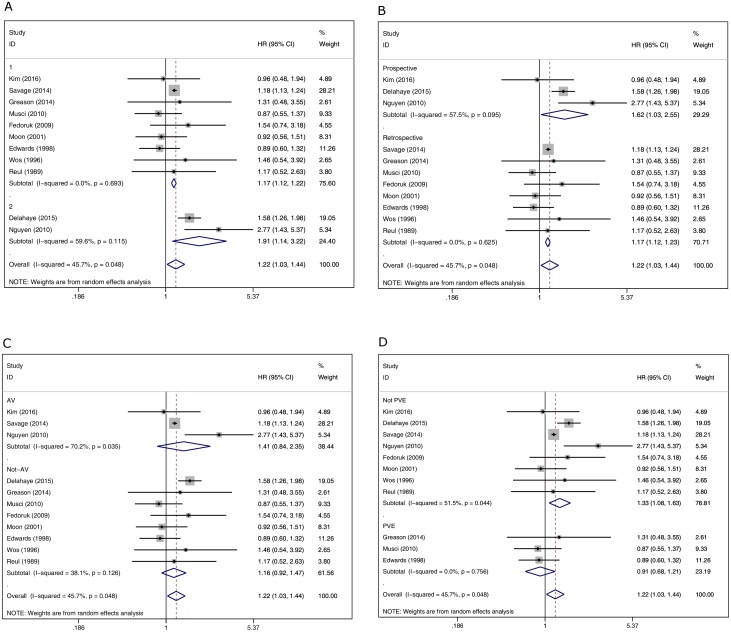
(A) Subgroup analysis of nine studies in the interval. (B) Subgroup analysis of study design. (C) Subgroup analysis of patients with isolated aortic valve endocarditis. (D) Subgroup analysis of patients with isolated PVE.

A Galbraith plot was used to detect the source of heterogeneity, and the results found that studies by Delahaye et al[4] and Nguyen et al[12] were located outside the interval. A subgroup analysis between the two studies outside the interval revealed the risk of death in the biological valve group was significantly higher than in the mechanical valve group(HR 1.91,95% CI 1.14 to 3.22, *P* = 0.015) (Fig 3). Heterogeneity was high among the included studies (I^2^ = 59.6%). Nine studies in the interval revealed the risk of death in the biological valve group was significantly higher than in the mechanical valve group (HR 1.17, 95% CI 1.12 to 1.22, *P* = 0.000) (Fig 4A). There was no heterogeneity among the nine studies (I^2^ = 0.0%). We found that I^2^ changed from 45.7% to 0.0% after the exclusion of the two studies, so the studies by Delahaye et al. and Nguyen et al. were considered as the source of heterogenity.

Because there were three prospective studies within the included studies, we divided the studies into a prospective study group and a retrospective study group to perform a subgroup analysis. In both the prospective study group and the retrospective study group, the risk of death in the biological valve group was significantly higher than in the mechanical valve group(HR 1.62, 95% CI 1.03 to 2.55, *P* = 0.038 and HR 1.17, 95% CI 1.12 to 1.23, *P* = 0.000, respectively) (Fig 4B). Heterogeneity was high among the prospective study group (I^2^ = 57.5%); however, there was no heterogeneity among the retrospective study group (I^2^ = 0.0%).

Only three studies reported isolated aortic valve endocarditis, and an analysis of these three studies indicated a higher HR of all-cause mortality in the biological valve group, but it was not statistically significant (HR 1.14, 95% CI 0.84 to 2.35, *P* = 0.192, I^2^ = 70.2%) (Fig 4C).

We performed a subgroup analysis of studies to determine whether biological valves had a better prognosis than the mechanical valve group in isolated PVE patients. The result showed a lower HR of all-cause mortality in the biological valve group compared to the mechanical valve group, without statistical significance (HR 0.91, 95% CI 0.68 to 1.21, *P* = 0.520, I^2^ = 0.0%) (Fig 4D). Only the study by Fedoruk et al.[9] incorporated patients with isolated native valve endocarditis among the eleven studies included. The study found that the survival of patients with biological valves was inferior to those with mechanical valves (HR = 1.54, 95% CI 0.74 to 3.18).

#### Recurrence of endocarditis

**Fig 5:**
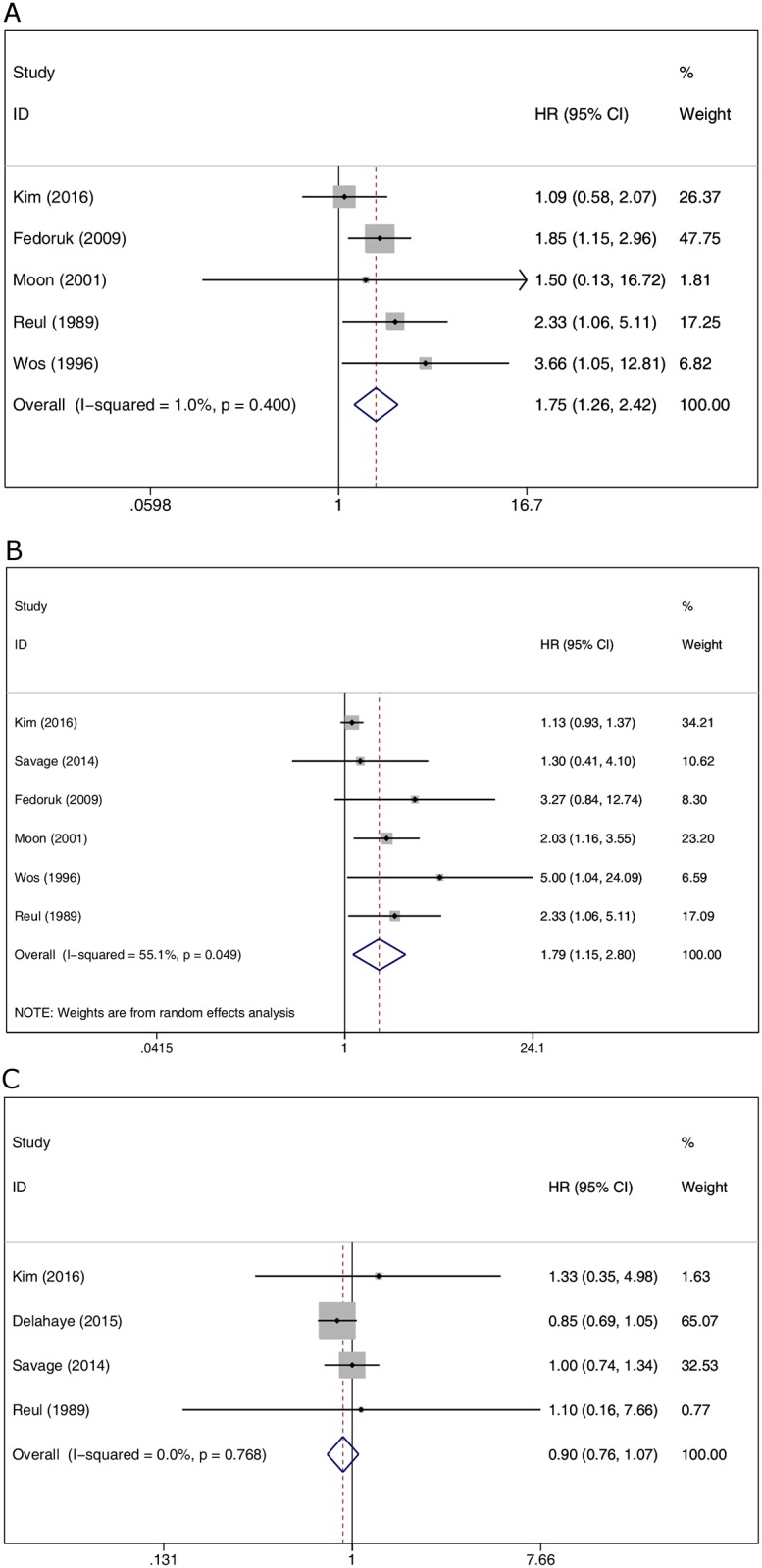
(A) Comparison of the recurrence of endocarditis between biological valves and mechanical valves. (B) Comparison of the incidence of reoperation between biological valves and mechanical valves. (C) Comparison of postoperative embolic events between biological valves and mechanical valves.

Five studies reported recurrence of endocarditis at follow-up (Fig 5A) and heterogeneity was low among the included studies (I^2^ = 1.0%). There was a statistically significant higher trend of relapse with biological valve group (HR 1.75, 95% CI 1.26 to 2.42, *P* = 0.001).

#### Reoperation

The incidence of reoperation was reported in six studies (Fig 5B). There was a statistically significant higher incidence of reoperation in the biological valve group (HR 1.79, 95% CI 1.15 to 2.80, *P* = 0.010). However, the heterogeneity was high (I^2^ = 55.1%).

#### Postoperative embolic events

Four studies reported postoperative embolic events (Fig 5C). Pooled RR did not show a significant difference in the risk of embolic events between the biological valve group and the mechanical valve group (RR 0.9, 95% CI 0.76 to 1.07, *P* = 0.245). There was no heterogeneity among the included studies (I^2^ = 0.0%).

### Publication bias

**Fig 6:**
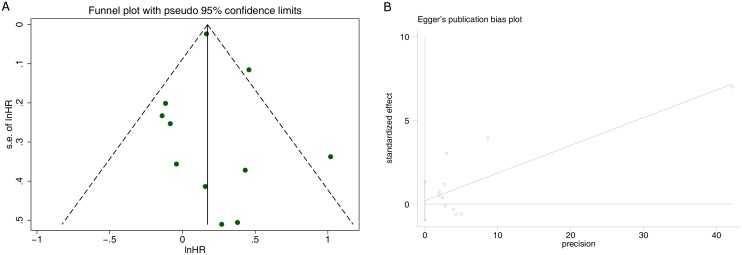
(A) Funnel plot of studies on all-cause mortality.(B) Egger’s regression test of studies on all-cause mortality.

Risk ratio (lnHR) results were used to produce a funnel plot; the result showed that both sides are symmetrical, and it could be concluded that the possibility of publication bias was small (Fig 6A). Egger’s regression test was used to test bias, and the result showed that the value of t was -0.38, *P* = 0.710>0.05, 95% CI -0.953275 to 1.343207. In the figure, we found the intercept across 0 points which indicated no statistical significance (Fig 6B).

## Discussion

Infective endocarditis (IE) is a fatal disease with a yearly incidence of approximately 3–10 per 100,000 people[15]. Overall, 40–50% of patients with IE require surgical treatment; the main choices for the valve replacememt are prosthetic valves and autografts or homografts[16]. The application of allografts and homografts, however, is limited by poor availability and difficult surgical techniques[3]. Since its initial report by Osler William in 1885, the epidemiology of IE has evolved from predominantly a disease of young adults with rheumatic heart valve disease to a disease affecting the elderly, and those with prosthetic valves and intra-cardiac devices[17]. A study from The Society of Thoracic Surgeons Adult Cardiac Surgery (STS ACSD) showed that, for primary operations, biological valve use increased from 57% to 67%, while mechanical valve use decreased from 30% to 24% during 2005 to 2011[11]. For reoperation, the use of biological valves increased from 38% to 52%, and the use of mechanical valves decreased from 20% to 17%[11]. We found that the utilization rate of biological valves was higher than mechanical valves in the primary operation and reoperation, but it was not clear whether biological valves can lead to a better prognosis compared to mechanical valves[18]. A meta-analysis from Lund et al. [19] including 17,439 patients suggested that there was no significant difference in the all-cause mortality between the biological valve group and the mechanical valve group after adjusting for age and other common risk factors. However, this meta-analysis was not aimed at patients with IE. Only 2.2% of the patients had IE in the biological valve group, and only 6.8% patients had IE in the mechanical valve group. Therefore, the results of this meta-analysis did not apply to patients with IE.

Our meta-analysis found that the risk of early mortality and all-cause mortality after surgery were 1.21 times and 1.22 times higher, respectively, in the biological valve group than in the mechanical valve group, which was statistically significant. Unfortunately, these data do not adjust for age, heart function and other basic factors. It was surprising that the results were not statistically significant after the exclusion of a study of Savage et al [11] with maximum weight.

The results of the retrospective study by Savage et al. [11] are consistent with our research, which suggested that the all-cause mortality of the biological valve group was 63.2% and the mechanical valve group was 53.4%, the HR was 1.18. The OR of the mechanical valve group and the biological valve group was 0.89, *P* = 0.142, which was not statistically significant after adjusting for risk factors. The study included 7,540 cases of patients with aortic valve endocarditis, including 5,396 cases in the biological valve group with an average age of 56 years and 2,144 cases in the mechanical valve group with an average age of 44.8 years; the heart function of the patients with stage III / IV was 57.3%, 53.1%, respectively. The conclusion was consistent with the prospective study by Kim et al., but was inconsistent with the prospective study by Delahaye et al.[4] and Nguyen et al.[12]. The data from Delahaye et al.[4] from the International Collaboration on Endocarditis—Prospective Cohort Study (ICE-PCS) suggested that biological valves were independently associated with a higher in-hospital and 1-year mortality (HR 1.298, *P* = 0.046), a result which is possibly more related to patient characteristics such as age (62 versus 54) than valve dysfunction. Another prospective study by Nguyen et al.[12] suggested that the 5-year mortality of the biological valve group was significantly higher than the mechanical valve group (adjusted HR 2.39, *P* = 0.029); however, this study only included 31 patients with biological valves who had a much older age (63.2 versus 57.3). Therefore, the results were not accurate. Moreover, the study dates were from a cross-sectional prospective population-based survey. We found that patients with biological valve were significantly older than those with mechanical valve, which may be the reason that the all-cause mortality of the biological valve group was slightly higher than that of the mechanical valve group. After all, age was a main factor affecting the survival of IE [9,12]. Another reason for this result may be the higher incidence of recurrence and reoperation caused by the degeneration of the biological valve[20].

A randomized trial has suggested that biological valves were more prone to dysfunction in patients < 65 years, while there was no significant difference in patients ≥ 65 years [20]. However, this study was not aimed at patients with IE. Two studies[4,12] including patients with IE suggested that the one-year and five-year mortality of patients with biological valves was significantly higher compared to patients with mechanical valves who were < 65 years of age, but there was no significant difference in patients ≥ 65 years of age. Moon and colleagues[10] reported that the long-term survival was similar in those who received a biological valve or mechanical in patients ≤ 60 years (52% at 15 years, 50% at 15 years). For patients > 60 years, the long-term survival tended to be higher in patients who received a biological valve (31% at 10 years, 17% at 15 years) compared with those who received a mechanical valve(18% at 10 years). However, the number of older patients who received a mechanical valve was small (15 of 87, 17%).

PVE is the most serious form of IE, occurring in 1–6% of patients with valve prostheses, and it has a 10–30% incidence of all cases of IE. It affects biological and mechanical valves equally[3,21,22]. In our study, all-cause mortality was not significantly different between the biological valve group and the mechanical valve group in patients with PVE, which was similar to findings by Musci et al.[8]. Musci and colleagues reported that the survival rates of 30 days, 1 year and 5 years after operation were 80%, 73.7%, and 53% in patients with biological valves and 67.2%, 50.7%, 36.9% in patients with mechanical valves. This study also suggested that the mortality rate of patients with mitral valve was significantly higher than that of patients with aortic valve, which may be the cause of the high HR in the study by Greason et al.[7]. In addition, the study of Greason et al. included only 39 patients.

Patients with IE had a higher risk of recurrent endocarditis, with an estimated 1.3% of patients per year, and Staphylococcus aureus infection was considered to be an important risk[23,24]. The optimal prosthesis has been debated for decades in the treatment of PVE[25]. Because of the advantages of removing the abscess, larger valve area, anti-infective and anti-coagulation therapy, surgical dogma indicate that a homograft or allograft is more beneficial than a prosthetic valve in the infected field[26]. However, the structural degradation and complicated surgical methods have limited their application [9,27]. A prospective study [26] from the databases of 2 tertiary academic centers suggested no difference in the prognosis of homograft and prosthetic valves in patients with IE. Our study suggested that there was a statistically significant higher trend of relapse in the biological valve group. This trend was largely influenced by the study by Fedoruk et al.[9], in which a univariate analysis indicated that the incidence of recurrence was 2.68 times higher in the biological valve group than in the mechanical valve group, which was statistically significant. However, the HR of the study changed to 1.89 with multivariate analysis in the general population. The author considered that this may be because 16.7% of the included patients were intravenous drug users or HIV patients, and most of them were treated with biological valves. Research has found that 30% of the cases of healthcare-associated IE were related with aseptic measures during venous catheter manipulation and during any invasive procedures[3]. Kim and colleagues[26] found no difference in the incidence of recurrence between the biological valve group and the mechanical valve group. The study by Moon et al[10] suggested that the rate of recurrent endocarditis during the first 5 years was 3.6% per patient-year in the mechanical valve group, and 2.1% per patient-year in the biological valve group. After 5 years the rate became 1.1% and 0.5% per patient-year, respectively, but the differences remained insignificant between groups. In addition, the cause of the high HR in the study by Wos et al.[7] may be because too few patients were included—only 17 patients with biological valves were included.

In our study, the incidence of reoperation was higher in the biological valve group, which may be due to the degradation of the biological valves. Moon and colleagues [10] suggested that the incidence of reoperation in the biological valve group (44% at 10 years, 78% at 15 years) was higher than in the mechanical valve group (26% at 15 years). Moreover, 63% of the biological valve reoperations were due to the degradation of the valve, and 35% were due to the recurrence of IE and parivalvular leakage. For patients with biological valves, the rate of reoperation was higher in patients ≤ 60 years (49% at 10 years) than those > 60 years (9% at 10 years). Fedoruk and colleagues[9] reported that the rate of reoperation in patients with recurrent PVE was 8.3% at 10 years, 14.2% at 15 years, 27.2% at 20 years, and 83.8% of the reoperations occurred in the biological valve group. The rate of reoperation for bleeding was slightly higher in patients with biological valves compared to mechanical valves[11].

In our study, the incidence of postoperative embolic events was slightly lower in patients with biological valve than in those with mechanical valves, although this difference was not statistically significant. Only four studies reported the incidence of embolic events, thus, strong conclusions could not be derived from these data. A prospective study [4] reported that the incidence of postoperative embolic events in patients with biological valves was lower than that in patients with mechanical valves, *P* = 0.0484. However, the embolic events occurring within one year after the operation do not reflect long-term effects in this study. Another larger retrospective study [11] reported that the incidence of postoperative embolic events was not different between biological valves and mechanical valves, which was 2.8% for both the 5,396 patients with biological valve and 2,144 patients with mechanical valve.

## Limitation

Our study has several limitations. First, our research consists of prospective or retrospective studies without randomized studies. Additionally, the patients in the biological valve group were inferior physical condition than those in the mechanical valve group, and the follow-up time of a study[4] was too short to completely reflect the prognosis of the patients. Moreover, several studies with small sample size raised some concerns regarding the reliability of their results [7, 8, 12, 28]. Second, the data were first selected from the literature, then extracted from the Kaplan-Meier curve, which may lead to bias. Third, individual studies mentioning prosthetic valve endocarditis, recurrence of endocarditis, reoperation and embolic events were limited in number.

## Conclusion

Results of our meta-analysis suggest that mechanical valves can provide a significantly better prognosis than biological valves in patients with infective endocarditis. There were significant differences in the clinical features of patients receive a biological valve compared to patients receive a mechanical valve. A largest, multicenter retrospective study included in our meta-analysis suggested that any mortality risk of the biological valve group was significant higher than that of the mechanical valve group, however, the risk was no different after risk adjusted. So, we thought the reason for this result may be related to the characteristics of the patient rather than valve dysfunction. Although several prospective studies have investigated this issue, the results were not consistent. Further randomized studies are necessary to verify the conclusions.

## Supporting information

S1 Fig Subgroup analysis of country. (TIF)

S1 Table PRISMA checklist. (DOC)

S2 Table Characteristics of patients included in the studies. (TIF)
