# Supplementary figures and images for: The prognosis of infective endocarditis treated with biological valves versus mechanical valves: A meta-analysis
Source: PLoS One. 2017 Apr 13;12(4):e0174519. doi: 10.1371/journal.pone.0174519 (PMC5390962; doi:10.1371/journal.pone.0174519)

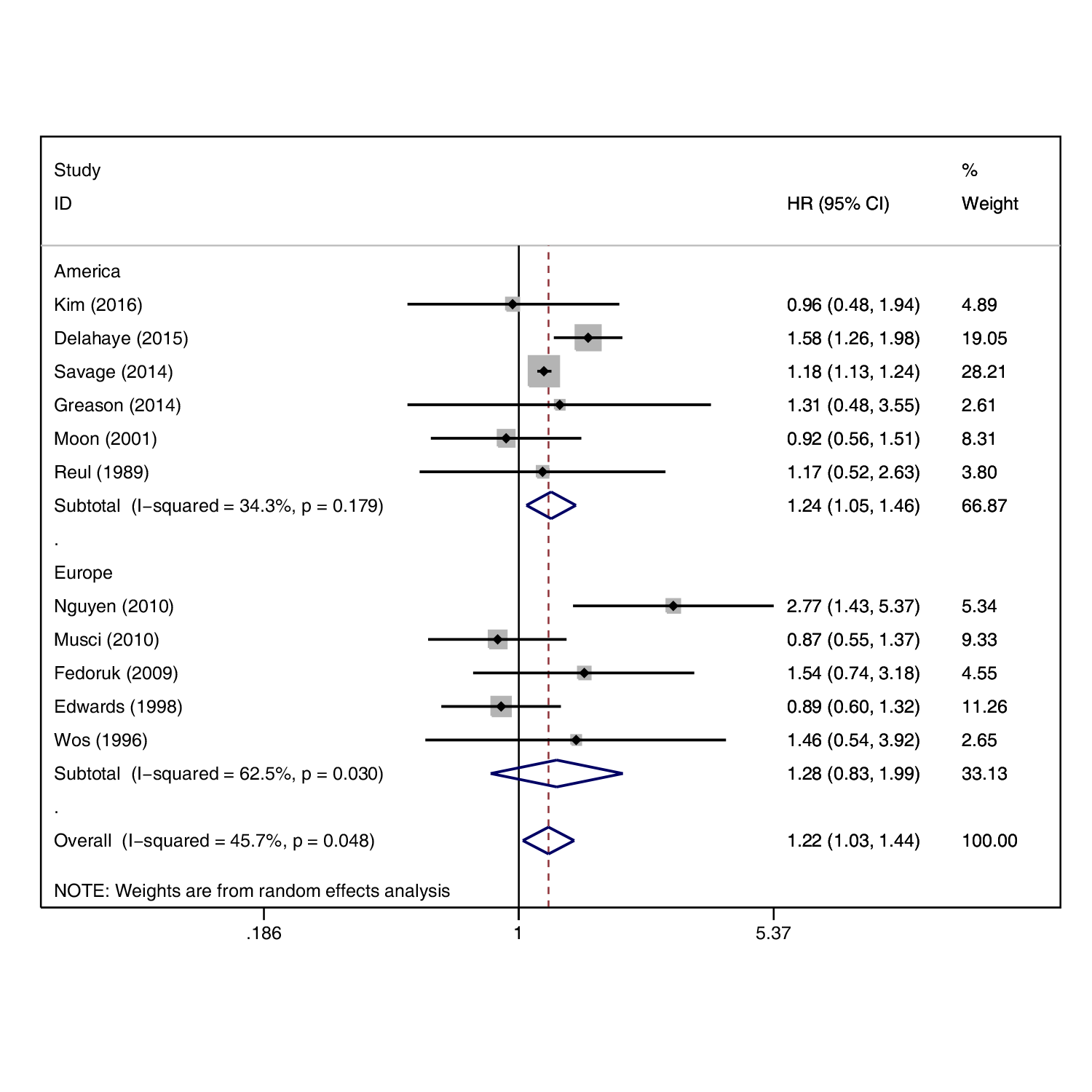

Supplement: S1 Fig — (TIF) [file pone.0174519.s001.tif]

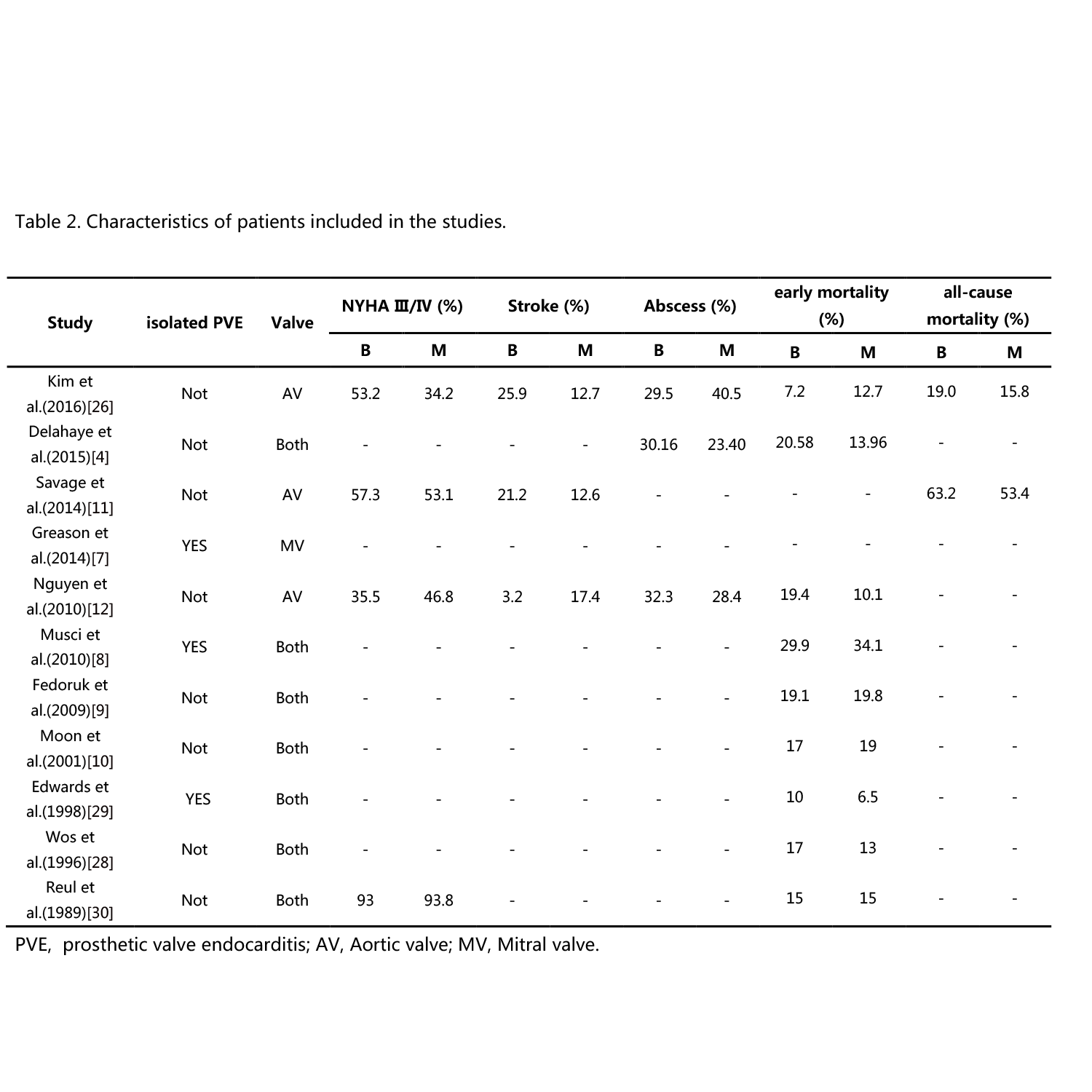

Supplement: S2 Table — (TIF) [file pone.0174519.s003.tif]
